# Nanoparticles from grape seed extract inhibit inflammatory cytokines and ameliorate CCl_4_-induced hepatotoxicity

**DOI:** 10.1186/s12906-025-05005-7

**Published:** 2025-07-19

**Authors:** Neveen Adel Madbouly, Doaa Mohammad Ali, Alyaa Ahmed Farid

**Affiliations:** 1 Zoology Department, Faculty of Science, Cairo University, Giza, 12613 Egypt; 2 Biotechnology Department, Faculty of Science, Cairo University, Giza, Egypt

**Keywords:** Muscat of Alexandria, Grape seed extract, Nanoparticles, CCl4-induced hepatotoxicity, Antioxidant, Anti-inflammatory

## Abstract

**Background:**

Xenobiotic-induced liver injury is a clinically reverent condition which may influence the development of steatohepatitis via affecting numerous pathways such as oxidative stress, inflammation, mitochondorial functioning and fatty acid biosynthesis. The current study was conducted to survey the antioxidant effect of grape seeds extract nanoparticles (GS extract NPs) against CCl_4_-induced oxidative stress, hepatic dysfunction and inflammatory changes.

**Methods:**

Hydroethanolic Grape seed (GS) extract was prepared and characterized using high performance liquid chromatography (HPLC), inductively coupled plasma–mass spectrometry (ICP-MS) and gas chromatography–mass spectrometry (GC-MS). Then, GS extract NPs were synthesized and in vitro antioxidant, anticoagulant, cytotoxicity and anti-inflammatory testes confirmed biological activity. Finally, Twenty-five male Sprague-Dawley rats were divided into five groups (*n* = 5/group). Either GS extract (200 mg/kg/day) or GS extract NPs (100 mg/kg/day) were orally administrated independently to CCL_4_-intoxicated (0.5 ml/kg twice a week for 3 weeks) rats. Four weeks after the treatment, serum levels of alanine aminotransferase (ALT), aspartate aminotransferase (AST), alkaline phosphatase (ALP) were monitored. In addition, hepatic Malondialdehyde (MDA), Superoxide dismutases (SOD), Glutathione (GSH) and catalase (CAT) and hepatic inflammatory cytokines were assessed.

**Results:**

GS extract NPs were spherical-shaped and regular particles (size: 16.5 to 22.5 nm and zeta potential: -39.42 mv). CCL_4_ -intoxicated rats showed increase of serum ALT, AST, ALP, elevation in MDA level accompanied by a decline in SOD, GSH and CAT levels in liver, compared with CCL_4_-untreated rats. Immunologically, serum C-Reactive Protein (CRP) and hepatic interleukin 1β (IL-1β), IL-4, IL-6 and Tumor Necrosis Factor-alpha (TNF-α) showed significant elevation, compared with CCL_4_ -untreated rats. Conversely, GS extract NPs supplementation potentially ameliorate hepatic functions by normalization of serum ALT, AST and ALP, reduced MDA level, improved antioxidant CAT, regulated liver inflammatory cytokines via maximal reduction of hepatic IL-1β, IL-4, IL-6 and TNF-α.

**Conclusion:**

GS extract NPs augmented the antioxidant and anti-inflammatory properties of GS extract thereby protecting the liver against oxidative stress induced by CCl_4_ as hepatic xenobiotic.

**Clinical trial number:**

Not applicable.

## Introduction

The liver is highly susceptible to damage by several exogenous compounds such as environmental pollutants, commercial products, chemicals and medications as it plays such an important role in storage, secretion, detoxifying and metabolic processes [1]. The chemical-induced acute and chronic liver injury is referred to as hepatotoxicity. Hepatotoxicity is multifaceted and can result from a range of pathways, including bioactivation, oxidative and cellular stress, mitochondrial and endoplasmic reticulum stress and more [2]. Drug-induced hepatotoxicity has emerged as a leading cause of acute liver disease, suggesting a potential therapeutic challenge over the last few decades and gaining considerable attention [3]. Carbon tetrachloride (CCl_4_) is one of the xenobiotics that frequently employed to trigger hepatotoxicity in animal models [4]. CCl_4_ hepatotoxicity is marked by hepatocellular necrosis and fat deposition [5]. When CCl_4_ level exceed the liver’s regenerating capability, severe liver failure frequently develops [6]. Higher dosages of CCl_4_ elicit nonspecific toxicity, leading to central nervous system depression and respiratory failure, which can result in death [7]. During CCl_4_ metabolism by cytochrome P-450 in the liver, free radical production promotes lipid peroxidation, covalent bonding of radicals to structural proteins, nuclear and mitochondrial DNA, and DNA aberration [8]. Furthermore, CCl_4_ promotes the liver’s resident macrophages, known as Kupffer cells, which generate and release chemo-attractants and neutrophil activators, resulting in neutrophil migration, production of reactive oxygen species and promote inflammatory alterations in hepatocytes, resulting to hepatotoxicity [9].

Considering substantial advances in modern medicine, management of liver disorders that can totally promote hepatic function, protect the organ, or restore hepatic cells remains a challenge. Several plants have been investigated for antioxidant, anti-inflammatory, and hepatoprotective properties in rats and mice. Research efforts have found that the main contributors for their beneficial properties are an abundance of phytochemicals including, phenols, essential oils, alkaloids, flavonoids, organic acids, coumarins, lignans, monoterpenes, glycosides and carotenoids [10–12].

The grape (*Vitis vinifera*, ‘Muscat of Alexandria’) is thought to have originated in North Africa before spreading around the Mediterranean from the port of Alexandria, Egypt, probably during the Roman Empire. Muscat of Alexandria has versatile variations; it is utilized as a table grape in Spain, Italy, Japan, and South America, as well as for dessert wine and blending in southern Europe, Africa, and South America [13, 14]. Muscat of Alexandria was recommended by several studies as “a drug-food” due to the medicinal properties of its active ingredients that present in fruit and leaves [15]. Several investigations have focused on the health-promoting, antioxidant, anti-inflammatory, hypocholesterolemic, immunological stimulant properties as well as chemopreventive activity against cardiovascular disease, pulmonary fibrosis and some cancers of grapes [16–20]. Grape seeds were also identified to contain substantial amounts of a variety of vitamins, 35% fibers, 13% fats, 11% proteins, 7% water and 3% minerals [21]. In addition to, high phenolic and essential fatty acid content that has sparked a new interest in their health benefits. Grape seed contain the majority of phenolic compounds, including catechin, epicatechin, gallic acid and procyanidins [22]. Polyphenol-rich grape seed (GS) extract has shown both in vitro and in vivo anti-inflammatory and antioxidant activities [23–25]. GS extract with proanthocyanidins surpassed vitamin C, E, and β-carotene in its antioxidant capacity [26].

Now, GS extract is available in several forms, including powder, liquid, pills, and tablets as dietary supplement. Additionally, the discovery of GS extract bioactive effects was extended to human healthcare researches. GS extract has been found to possess anti-obesity potential [27], reduce the risk of vascular disease in patients with diabetes mellitus [28], diabetic nephropathy [29] and diabetic hepatopathy [30]. Furthermore, GS extract show selective antitumor activities towards oral cancer cell lines (CAL27 and SCC25) [31], colorectal cancer in HT-29 cells [32] and A431 skin cancer cells [33]. GS extract has been demonstrated to be safe in clinical trials up to 8 weeks but a side effect profile for GS extract supplements may develop as symptoms of itchy scalp, nausea, disorientation, vomiting, indigestion, diarrhea, headache, sore throat, cough, and rash after long-term consumption [34–36]. GS extract may also affect the pharmacokinetics of blood thinners, warfarin, cytochrome P450 3A4 and UGT substrate medications [37]. Moreover, in the gut, the its digestion was suggested to affect the stability, composition and inhibit the anti-inflammatory activities of GS extract [38, 39]. The bioaccessibility of GS polyphenols is influenced by pH and digestive enzymes conditions [40] so that efforts should be focused now on increasing the bioavailability of the bioaccessible polyphenols obtained from GS extract.

In order to improve the GS extract bioavailability, stability and reduce the release of bioactive compounds nanotechnology could be beneficial. Different nanoencapsulation techniques were studied in previous research including, liposomes [41], chitosan encapsulation [42], zinc oxide nanoparticles [43] and poly(lactic-co-glycolic) acid nanoparticles (PLGA NPs) [44]. Based on previous reports [45, 46], grape seed nanoparticles (GSNPs) can be prepared in a novel method using methanol and 38% hydrogen chloride (HCl). As a first attempt to highlight the efficacy of GS extract NPs prepared in methanol and HCl, the present study aims to assess the hepatoprotective and anti-inflammatory potential of GS extract compared with its nanoparticle formulation (GS extract NPs) against CCl_4_-induced acute hepatotoxicity, with a focus on antioxidant defense, and cytokine-mediated inflammation.

## Methods

### Chemicals and kits

The analytical standards for high-performance liquid chromatography (HPLC), Inductively coupled plasma–mass spectrometry (ICP-MS), Gas chromatography–mass spectrometry (GC-MS) systems, 1, 1-Diphenyl-2-Picryl Hydrazyl (DPPH) and MTT (3-[4, 5-dimethylthia- zol-2-yl] − 2, 5-diphenyl tetrazolium bromide) were procured from Sigma-Aldrich Co. (St Louis, USA). Alanine aminotransferase (ALT), aspartate aminotransferase (AST) and alkaline phosphatase (ALP) using Elabscience^®^ (Texas, USA). Malondialdehyde (MDA) level and the antioxidant enzymatic activity of SOD, Superoxide dismutases (SOD), Glutathione (GSH), catalase (CAT) and C-Reactive Protein (CRP) using Elabscience^®^ (Texas, USA). Interleukin 1β (IL-1β), IL-4, IL-6 and Tumor Necrosis Factor-alpha (TNF-α) obtained from MyBioSource Inc. (San Diego, USA). All other chemicals and reagents used during the tests were of the highest analytical grade available.

### Formulation of grape seeds (GS) extract

*Vitis vinifera* “Muscat of Alexandria” fruits were collected from a Faculty of Agriculture, Cairo University campus, and the formal identification of this plant was carried out by Prof. Dr. Rim Hamdy, a member of Cairo University’s Herbarium and Professor of Taxonomy and Flora in the Department of Botany at Faculty of science, Cairo University. The specimen was identified as *Vitis vinifera* “Muscat of Alexandria” (Family: Vitaceae), using floral keys to compare the species gathered for the study with verified specimens housed in Cairo University Herbarium (CAI) (Voucher number: cai.63.2741.23).

After collection, GS were dried at room temperature, milled into powder and then weighed. A hydroethanolic extract of 100 g GS powder was performed using water and ethyl alcohol as solvents by ratio water: ethyl alcohol (40: 360 ml) for 24 h under continuous stirring. The extracted material was filtered using Whatman No. 1 filter paper and concentrated using a rotating vacuum evaporator at 40 °C, ethanol fraction was evaporated and the aqueous GS extract was then collected, filtered and lyophilized. The dried GSE was collected, weighed and stored at 4 °C until further use [46–49]. GSE yield was evaluated according to Equation.

Yield (%) =$$\:\frac{mass\:of\:grape\:seed\:extract}{mass\:of\:initial\:grape\:seed\:powder}$$ × 100

### Formulation of GS extract NPs

Grape seed powder (500 mg) was mixed with 38% hydrogen chloride (3–5 ml) and methanol (30 ml) in a Scott bottle. The mixture was placed on a magnetic hotplate stirrer (Boeco MMS-3000, Boeco, Germany) adjusted to temperatures of 30° C at 3000 rpm. After two hours of vigorous stirring, distilled water (30 ml) was added followed by continuous stirring for extra two hours. After this step, the mixture was then centrifuged for 15 min at 9000 rpm and filtered. Then the supernatant was collected, dried in oven at 50–60° C for 12 h to get GS extract NPs in powder form and weighted. pH was adjusted to 7 by washing of GS extract NPs several times with deionized water, dried again then stored at -20° C till further analysis [45, 46, 50].

Yield (%) =$$\:\frac{mass\:of\:grape\:seed\:extract\:NPs}{mass\:of\:initial\:grape\:seed\:extract}$$ × 100

### Characterization of GS extract and GS extract NPs

#### High performance liquid chromatography (HPLC) analysis of GS extract and GS extract NPs

Adopted HPLC system was Agilent 1260 Infinity (Aglient Technologies, Palo Alto, CA, USA). The chromatographic separation of the GS extract was accomplished using an Agilent Eclipse 40 °C Eclipse C18 column (4.6 mm × 250 mm i.d., 5 m, Santa Clara, CA, US). The mobile phase was a mixture of (A) water and (B) acetonitrile/trifluoroacetic acid (0.05%). The following gradient runs were planned: 0 min (82%A); 0–5 min (80%A); 5–8 min (60%); 8–12 min (82%A); 15–16 min (82%A); and 16–20 min (79%A). The column was maintained at a constant 40 °C. The sample’s injection volume was 5 µL. The UV detector is set to detect wavelengths of 280 nm.

#### Inductively coupled plasma–mass spectrometry (ICP-MS) analysis of GS extract and GS extract NPs

Metals in GS extract and GS extract NPs samples were analyzed using Agilent 7900 ICP-MS (Agilent Technology, Santa Clara, CA). Briefly, about 250 mg of GS power was digested using oxidant mixture (1 ml H_2_O_2_ + 2 ml HNO_3_ + 3 ml H_2_O). The mixture was heated in digesting equipment with a program of three steps according to the following temperature-programmed settings: 1] from room temperature to 140 °C for 10 min with a hold for 1 min; 2] 190 °C for 4 min and hold for 5 min; 3] 190–270 °C for 5 min and hold for 10 min. For every sample, the digestion process was carried out three times. Upon the end of the digestion process, the sample solution evaporated at 140 °C for 20 min. After that sample was cooled and transferred to polypropylene flasks and added deionized water to make up to 10 ml [51, 52].

#### Gas chromatography–mass spectrometry (GC-MS) analysis of GS extract and GS extract NPs

A Trace GC1310-ISQ gas chromatography system (Thermo Scientific, Austin, TX, USA) coupled with a mass spectrophotometer was exploited to evaluate volatile composition of GS extract and GS extract NPs. The chromatography system was fitted with the TG-5MS direct capillary column (30 m × 0.25 mm × 0.25 μm film thickness). The temperature of the injector was 250 °C. After being held at 100 °C for one minute, the temperature was raised to 260 °C at a rate of 4 °C, and it was then held there for ten minutes. It was 250 °C in the detector. 1 mL/min of constant-flow helium gas was utilized as the carrier gas [53].

### Size, shape and zeta potential of GS extract NPs

Using Transmission Electron Microscope (TEM) (JEM-1400 Flash, JEOL, Watchmead, UK) with a copper mesh as support, the size and shape of the manufactured GS extract NPs TEM lab FA-CURP, Faculty of Agriculture, Cairo University -Research Park (CURP). Using the Dynamic Light Scattering (DLS) method, the hydrodynamic size of the formulated GS extract NPs was also determined in PBS and zeta potential (ZP) was detected (Zetasizer Nano ZS90; Malvern Instruments Limited, Malvern, UK).

### In vitro antioxidant ability of GS extract and GS extract NPs

1, 1-Diphenyl-2-Picryl Hydrazyl (DPPH) scavenging activities of GS extract and GS extract NPs formulation were monitored using ascorbic acid as reference control [54]. Serial dilutions of GS extract NPs and GS extracts (from 1000 to 1.95 µg/ml) were mixed with 1 ml free radical DPPH (0.1 mM) in methanol. The mixture was shaken vigorously and incubated in dark for 30 min at room temperature. The antioxidant activity of GS extract and GS extract NPs was established in three samples for each concentration. The reduction in DPPH free radicals was detected at 517 nm and the inhibition activity was calculated according to the following equation and expressed as mean ± standard deviation (SD) values:

$$\begin{aligned} & DPPH\;inhibition\;\% \\ & =\frac{([ Absorbance \;(control)-Absorbance\;(Sample)])}{(Control\;absorbance)} \times 100 \end{aligned}$$

### In vitro anticoagulant ability of GS extract and GS extract nps, prothrombin time (PT) and activated partial thromboplastin time (APTT) tests

PT and APTT tests were performed using different concentrations of GS extract and GS extract NPs (25, 50 and 75 µg/ml). 10 µl of GS extract, GS extract NPs or heparin (as control) was mixed with 90 µl of rat plasma and incubated for 30 min at 37^◦^C [55]. An ACL 200 coagulation analyzer (Diamond Diagnostics Inc., Bedford, MA, USA) was used to record the clotting time. The procedure was repeated three times for each sample, results presented using mean ± standard deviation (SD) values.

### In vitro cytotoxicity ability of GS extract and GS extract NPs

MTT (3-[4, 5-dimethylthia- zol-2-yl] − 2, 5-diphenyl tetrazolium bromide) assay was applied to estimate cell viability according to Kong et al. [56]. Briefly, under sterilized conditions rat hepatic cells were perfused and isolated according to Shen et al. [57] detailed protocol. The isolated hepatocytes were then filtered through nylon filter with 100 μm pore size mesh after that, hepatocytes were cultured onto plates for 4 h in William’s complete Medium (Thermo Fisher Scientific, MA USA), washed and counted. Cells were seeded in 24 well plates, at a density of 5 × 10^5^ cells/well with and incubated overnight in a humidified atmosphere (37 °C, 5% CO_2_). After 24 h, medium was replaced with HepatoZYME-SFM (Thermo Fisher Scientific, MA USA) and 100 µl/well of different GS extracts or GS extract NPs concentrations (ranging from 1000 to 31.25 µg/mL). After incubation for additional 24 h, 10 µl of MTT (final concentration 0.5 mg/ml) (Sigma Chemical, St Louis, MO) was added to each well and incubated for 4 h in a humidified atmosphere (37 °C, 5-6.5% CO_2_). Then 100 µl of the solubilization solution was added into each well and kept overnight in incubator till the complete solubilization of the purple formazan crystals. At 590 nm, the absorbance was detected using a multipurpose microplate reader. The cell viability was compared with normal untreated control. The IC50 value was computed. The trials were conducted in triplicate, and the cell viability was assessed using mean ± standard deviation (SD) values according to the following equation:

**Cell inhibition (%) = 1- [ Absorbance (test)/ Absorbance (control)] × 100**.

### In vitro anti-inflammatory ability of GS extract and GS extract NPs

the anti-inflammatory effects of GS extract and GS extract NPs were tested using membrane stabilization assay. RBCs hemolysis was assessed according to method. Rat blood was pipetted into heparin tubes and centrifugated 10 min on 3000 rpm to separate RBCs, washed and diluted as 40% suspension in PBS. GS extract and GS extract NPs were prepared different concentrations (from 100 to 1000 µg/mL). 0.15 ml of RBCs (40%) and 4.85 ml of isotonic buffer solution were mixed with GS extract, GS extract NPs or Indomethacin as reference (200 µg/ml), then kept for 20 min in a water bath at 54 °C and finally, centrifuged for 7 min at 3500 rpm. In the supernatant, the release of hemoglobin quantity was estimated at 560 nm absorbance [58]. Percentage of hemolysis inhibition was calculated from the equation:

$$\begin{aligned} & Hemolysis\;inhibition\;percentage\;(\%) \\ & = 1 - (OD\;sample - OD\;negative\;control)/ \\ & \quad (OD\;positive\;control - OD\;negative\;control) \\ & \times 100 \end{aligned}$$

### Animals and experimental design of in vivo study

Male, 180–200 g, 7–8 weeks old Sprague-Dawley rats were enrolled. Rats were purchased from the National Research Centre’s animal house colony in Cairo, Egypt. Animals were housed in adequate laboratory settings (60% air humidity, 22 ± 2 °C, 12-h light–dark cycle) for a week for adaptation. Standard pellet food and water were provided to the animals. All procedures were conducted in accordance with requirements of the Faculty of Science’s Institutional Animal Care and Use Committee (IACUC) with consent number (CUIF/24/23).

CCl_4_-induced hepatotoxicity was induced in rats by intra-peritoneal (i.p.) administration of CCL_4_, at a dose of 0.5 ml/kg dissolved in olive oil (1:1) (100 µl) twice a week for 3 successive weeks [59, 60]. Twenty-five rats were divided into five groups (*n* = 5/group). The experimental design was as follows: Group1: healthy control (HCs), rats received normal saline; Group 2: vehicle control that received olive oil only; Group 3: (positive control) CCL_4_ -intoxicated, untreated rats; Group 4: (CCL_4_ -GSE) rats were CCL_4_ -intoxicated for 3 successive weeks, then received GS extract (200 mg/kg/day) by oral gavage for 4 weeks; Group 5: (CCL_4_ -GSENPs) rats were CCL_4_ -intoxicated for 3 successive weeks, then received GS extract NPs (100 mg/kg/day) by oral gavage for 4 weeks. At the end of experimental time (7 weeks), all the rats were euthanized by decapitation under light anesthesia [Combination of ketamine (87 mg/kg) + Xylazine (13 mg /kg)]. Blood samples were collected in heparinized tubes from all animals. Livers were excised, washed in ice cold PBS and divided to prepare samples for hepatic tissue homogenate (for oxidative stress and cytokine levels estimation) and histopathological studies.

### Preparation of Sera and hepatic tissue homogenates

Sera were collected from blood samples after centrifugation (3000 rpm for 10 min at 4 °C). After homogenizing the liver tissues in PBS buffer, Tissue homogenates were generated in 1.0 ml of PBS/100 mg of tissue. Then were centrifuged for 15 min at 4 °C at 13,000 rpm then the supernatant from the homogenates were obtained.

## Determination of liver function parameters

Alanine aminotransferase (ALT), aspartate aminotransferase (AST) and alkaline phosphatase (ALP) activities were measured in serum using Elabscience^®^ (Texas, USA) commercial, colorimetric kits (Catalog number: E-BC-K235-S, E-BC-K236-S and E-BC-K091-S; respectively).

### Determination of oxidative stress parameters

Malondialdehyde (MDA) level and the antioxidant enzymatic activity of SOD, Superoxide dismutases (SOD), Glutathione (GSH) catalase (CAT) in the liver homogenate were estimated using commercial kits and according to manufacturer manual (Elabscience^®^, Texas, USA). MDA: competitive ELISA, Cat. No.: E-EL-0060. SOD: competitive ELISA, Cat. No.: E-BC-K020-M. GSH: colorimetric method E-BC-K030-S and CAT: colorimetric method, Catalog number: E-BC-K031-M].

### Determination of C-Reactive protein (CRP)

Serum CRP was measured using commercial sandwich ELISA (Catalog number: E-EL-R0506, Elabscience^®^, Texas, USA).

### Determination of inflammatory cytokines

Measurements of inflammatory cytokines interleukin 1β (IL-1β), IL-4, IL-6 and Tumor Necrosis Factor-alpha (TNF-α) in liver homogenates were analyzed using (MyBioSource, Inc., San Diego, USA) (Catalog numbers: MBS2023030, MBS2023426, MBS2021530 and MBS700574; respectively) all measurements were according to the manufactural instructions.

### Statistical analysis

The sample size in each group (*n* = 5) was determined based on statistical power analysis (G*Power software, SAS, UCLA) [61]. Test of the normality was applied using the Shapiro-Wilk test [62]. The collected data were presented as mean ± SD. using The Statistical Package for Social Services version 22 (SPSS) (IBM, Inc., Chicago, IL, USA) [63], the statistical significance of the difference was established using one-way ANOVA followed by post-hoc comparisons using Turkeyʼs multiple comparison test. A p-value < 0.05 was considered statistically significant.

## Results

### HPLC-identified polyphenols in GS extract and GS extract NPs

**Fig. 1:**
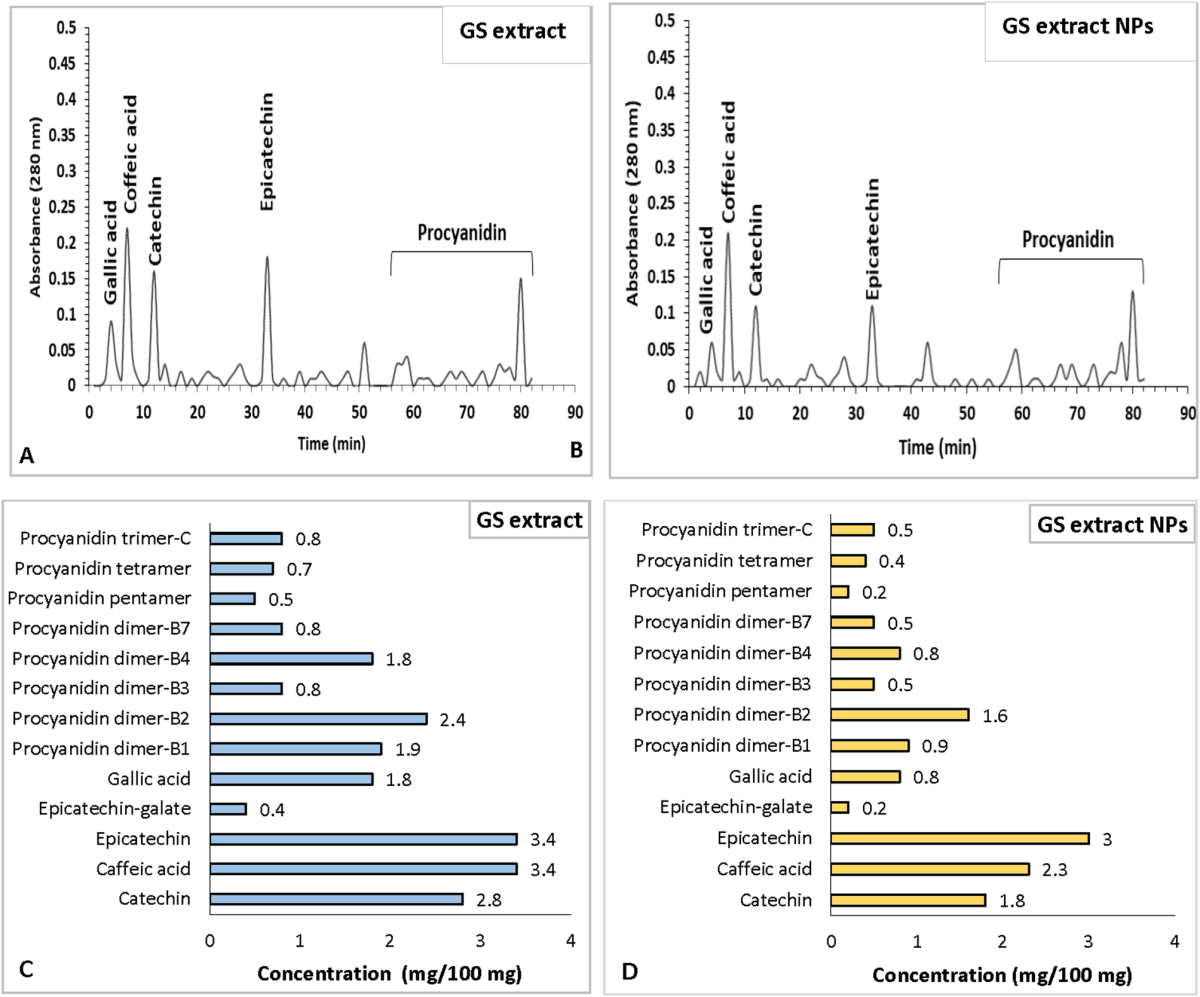
High Performance Liquid Chromatography (HPLC) profile of (**A**) grape seed (GS) extract and (**B**) grape seed extract nanoparticles (GS extract NPs) (**B**). polyphenolic concentrations (mg/100 mg) in (**C**) GS extract and (**D**) GS extract NPs

The hydroethanolic extraction of 1oo g of grape seed powder yield 8.39 g dry GS extract (8.39% yield). While GS extract NPs yield was 75.6%. The major prominent phenolic compounds were detected with HPLC in both GS extract (Fig. 1A) and GS extract NPs (Fig. 1B), including; gallic acid (4.22 min), caffeic acid (7.21 min), catechin (12.5 min), epicatechin (31.3 min) and procyanidins (54–83 min). The concentrations (mg/100 mg) of the polyphenolic contents of the GS extract (Fig. 1C) and GS extract NPs (Fig. 1D) were analyzed and various active polyphenol flavonoids (including catechin, epicatechin, epicatechin-gallate, procyanidin (dimer-B1, B2, B3, B4 and B7, trimer C, tetramer and pentamer) were detected. In addition to, bioactive phenolic acids (caffeic acid and gallic acid). GS extract sample contained higher polyphenols concentrations than GS extract NPs.

### ICP-identified elements in GS extract and GS extract NPs

According to ICP analysis of GS extract and GS extract NPs, calcium was the most abundantly detected element (5.8 and 4.5 mg/g, respectively) followed by potassium (3.6 and 2.8 mg/g, respectively). Lower content of magnesium, zinc, iron and other elements were illustrated (Table 1).

**Table 1:** Comparison of element’s concentration in grape seed (GS) extract and grape seed nanoparticles (GS extract NPs) by inductively coupled plasma (ICP)

Element	Concentration (mg/g)	
	GS extract	GS extract NPs
Calcium (Ca)	5.8	4.5
Potassium (K)	3.6	2.8
Magnesium (Mg)	1.3	0.8
Zinc (Zn)	0.9	0.2
Iron (Fe)	0.4	0.1
Phosphors (P)	0.2	0
Sodium (Na)	0.1	0
Manganese (Mn)	0.02	0
Aluminum (Al)	0.02	0
Copper (Cu)	0.002	0

### GC-MS-identified components in GS extract and GS extract NPs

The percentage of volatile components of GS extract and GS extract NPs according to GC-MS chromatography. We observed aldehydes, (such as benzoic and Cinnamon). In addition, a series of alcohols of the terpenes, like: borneol, carvacrol, geraniol, linalool and α-terpineol were also detected by GC-MS. Besides, various types of flavonoids, catechin, epicatechin, Myricetin, Kaempferol. Resveratrol is also detected as a stilbenoid. Polyphenolic acids were also present e.g. ellagic acid, gallic acid. Numerous essential and non-essential fatty acids, such as linoleic acid, linolenic acid, hexadecenoic acid, Oleic acid and palmitic acid were also found **(**Table 2).

**Table 2:** Major active component’s percentage (%) in in grape seed (GS) extract and grape seed nanoparticles (GS extract NPs) by GC-MS

Compound	Percentage (%)			
	GS extract		GS extract NPs	
Benzoic aldehyde	2.6	2.1		
Borneol	3.8	3.5		
Carvacrol	2.8	2.2		
Catechin	3.9	3.4		
Cinnamon aldehyde	3.4	3.2		
Ellagic acid	5.4	4.6		
Epicatechin	4.1	3.6		
Gallic acid	5.2	4.6		
Geraniol	6.2	6.1		
Hexadecenoic acid	7.8	6.8		
Kaempferol	8.9	8.1		
Linalool	2.7	2.3		
Linoleic acid	5.9	3.3		
Linolenic acid	0.7	0.2		
Myricetin	9.6	9.1		
Octadecanoic acid	5.1	4.8		
(9Z)-Octadecenoic acid	2.2	1.7		
Oleic acid	6.5	6.1		
Palmitic acid	3.9	3.1		
Quercetin	7.8	7.1		
Quencetin-3-monoglucoside	1.2	0.6		
Quercetin-3-monoglucuronoside	0.9	0.2		
Resveratrol	3.7	3.1		
Xanthosine	3.8	3.4		
α–terpeniol	2.8	2.2		
α-caryophyllene	1.9	1.1		

### Morphological characterization of GS extract NPs

**Fig. 2:**
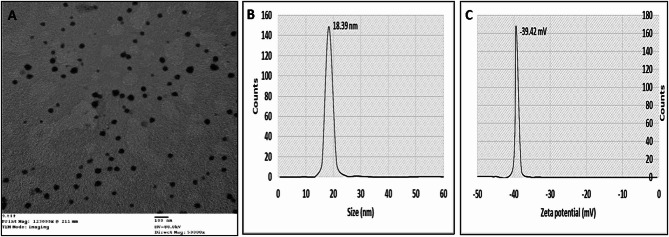
**(A)** The transmission electron microscopy (TEM) image of grape seed extract nanoparticles (GS extract NPs), Dynamic light scattering (DLS) analysis showing (**B**) size distribution (nm), (**C**) zeta potential (mV) of GS extract NPs

**Fig. 3:**
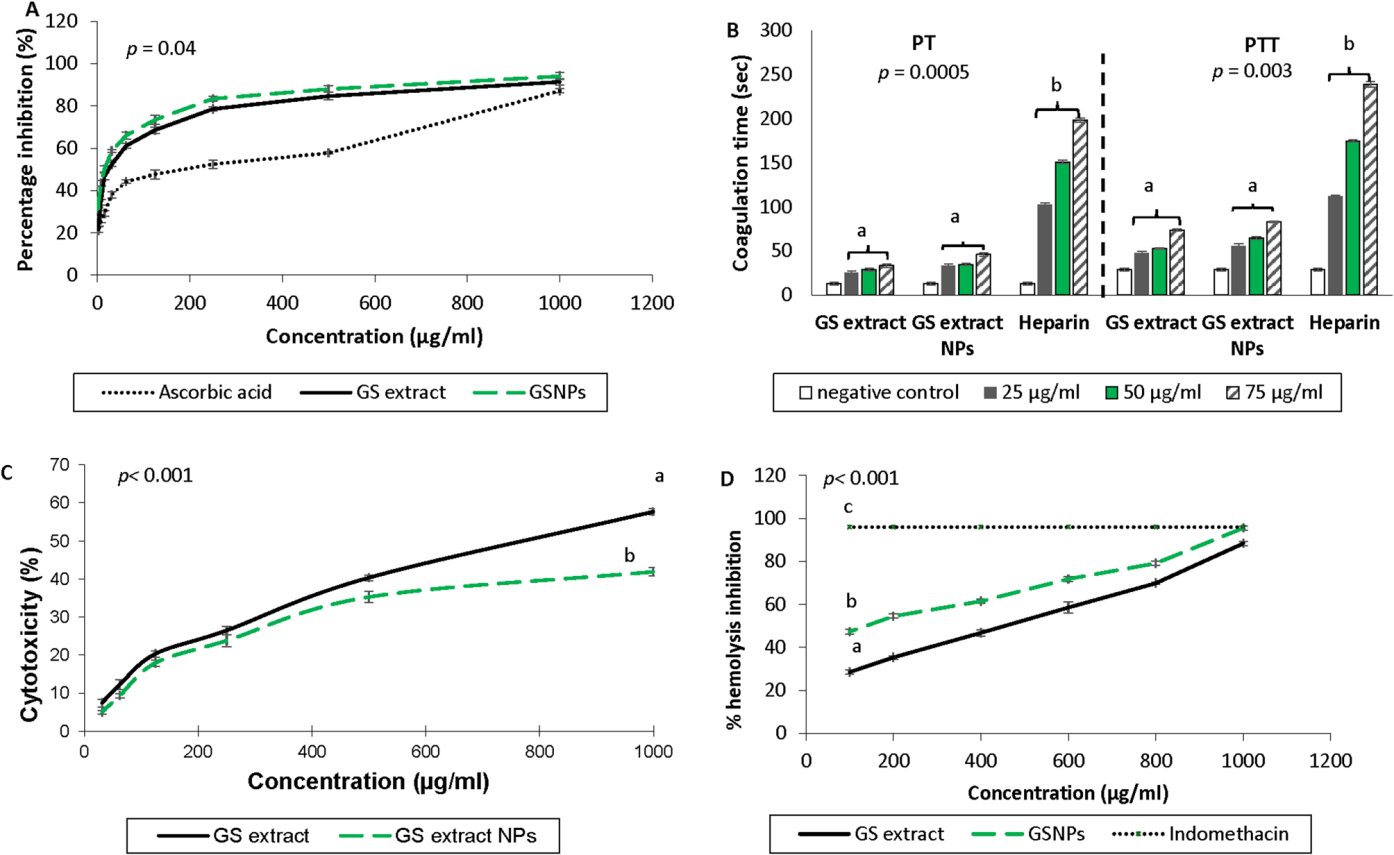
In vitro characterization of grape seed (GS) extract and GS extract nanoparticles (NPs). (**A**) The in vitro DPPH scavenging activity, (**B**) The anticoagulant activity, (**C**) In vitro MTT cytotoxicity assay and (**D**) The in vitro anti-inflammatory activity. Statistical analysis was carried out by one-way ANOVA followed by post-hoc Turkeyʼs test. Values marked with the same letter are not significantly different (*p* ≥ 0.05) whereas those marked with different ones are significantly differed (*p* < 0.05)

For morphological assessment, a transmission electron microscopy (TEM) analysis was performed, and the images are presented in Fig. 2A. GS extract NPs showed nearly spherical-shape and regular particles with size heterogeneity is observed (16.5 to 22.5 nm), Also, the hydrodynamic diameter of the GS extract NPs was measured using the DLS method. The diameter of GS extract NPs was found to be 18.39 nm in an aqueous medium, (Fig. 3B). The zeta potential of the prepared GS extract NPs was − 39.42 mv (Fig. 2C).

### Determination of in vitro antioxidant ability of GS extract and GS extract NPs (DPPH assay)

As shown in Fig. 3A, the antioxidant activity of GS extract NPs ranged from 31.5 to 94.16%, whereas the GS extract ranged from 21.6 to 91.5% at concentration range from 1.95 to 1000 µg/ml. It can be observed that the increasing concentrations of both GS extract and GS extract NPs from 1.95 to 1000 µg/ml significantly enhanced the antioxidant activity, compared with ascorbic acid as a standard. There is no significant difference (*p* ≥ 0.05), based on the antioxidant activity, between GS extract and GS extract NPs.

### Determination of in vitro anticoagulant ability of GS extract and GS extract NPs (PT and APTT test)

PT and PTT test were performed with different concentrations (25, 50 and 75 µg/ml) of GS extract, GS extract NPs and heparin. PT and PTT was significantly affected by gradual increase of concentrations of either GS extract, GS extract NPs and heparin (*p* < 0.00001).

The PT was significantly prolonged due to both GS extract (25.61 ± 1.41, 28.93 ± 1.61 and 33.53 ± 2.01 s, respectively) and GS extract NPs (33.6 ± 1.96, 34.86 ± 1.41 and 46.23 ± 2.16 s, respectively), compared with the negative control (13.03 ± 0.21 s) (*p* = 0.0005). The PTT was also increased with GS extract (48.21 ± 1.17, 52.91 ± 1.12 and 73.93 ± 1.16 s, respectively) and GS extract NPs (56.16 ± 2.45, 64.73 ± 1.82 and 83.11 ± 0.82, respectively), compared with the negative control (29.11 ± 0.38 s) (*p* = 0.003). On the other hand, the influence of GS extract and GS extract NPs on PT and PTT are not significantly different from each other (*p* ≥ 0.05) (Fig. 3B).

### Determination of in vitro cytotoxicity ability of GS extract and GS extract NPs (MTT assay)

Cytotoxicity of GS extract and GS extract NPs was assessed on normal rat hepatocytes using MTT regent. Treatment of rat hepatocytes with GS extract and GS extract NPs showed a significant (*p* < 0.001) cell growth inhibition in dose-dependent manner. At low doses (31.25 and 62.5 µg/ml), GS extract NPs was significantly (*p* = 0.012 and 0.018, respectively) less toxic than GS extract. Furthermore, higher concentrations (500 and 1000 µg/ml, respectively) GS extract NPs developed more significant (*p* < 0.001) cytotoxicity tolerance (35.26 ± 1.5 and 41.9 ± 1.13%, respectively) effects compared with GS extract (40.33 ± 0.71 and 57.7 ± 0.87%, respectively). Data analysis of MTT assay exploited IC50 values (dose required for 50% inhibition) of GS extract (783.52 µg/ml) and GS extract NPs (1087.71 µg/ml) on rat hepatocytes (Fig. 3C).

### Determination of in vitro anti-inflammatory ability of GS extract and GS extract NPs (membrane stabilization assay)

Both GS extract and GS extract NPs showed significant (*p* < 0.001) concentration dependent in vitro anti-inflammatory activity on RBCs membranes. GS extract NPs showed significantly higher anti-inflammatory efficiency (*p* < 0.001) compared with GS extract as it recorded highest anti-inflammatory efficiency reached 95.56% at 1000 µg/ml while, GS extract showed 88.4% at the same concentration (Fig. 3D).

### Determination of in vivo effects of GS extract and GS extract NPs on liver functions of CCL_4_-induced hepatotoxicity

**Fig. 4:**
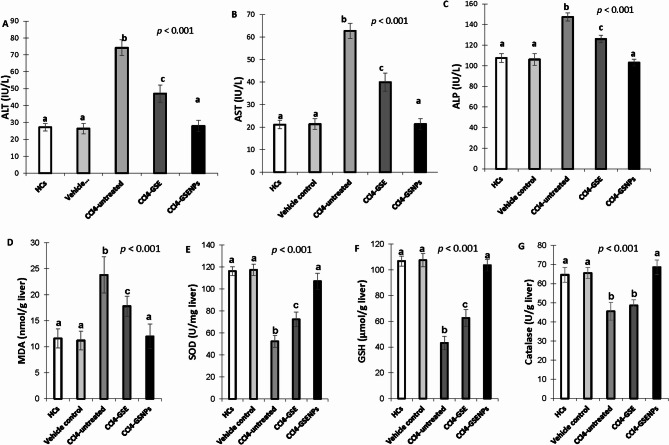
Biochemical and oxidative stress parameters in the different experimental groups. Levels of **A**] alanine transaminase (ALT), **B**] aspartate transaminase (AST), **C**] alkaline phosphatase (ALP) in serum, **D**] malondialdehyde (MDA) and E] Superoxide dismutases (SOD), F] Glutathione (GSH) and **G**] Catalase in liver homogenate measured following 30 days of treatment. HCs, negative control group; vehicle control, receives olive oil only; CCl_4_-untreated, liver injury rats that receive 100 µl CCL_4_, at a dose of 0.5 ml/kg, dissolved in olive oil twice a week for three successive weeks; CCl4-GSE, rats with CCL_4_-induced liver injury that receive grape seed extract (200 mg/kg/day) by oral gavage for four weeks; CCl_4_-GSENPs, rats with CCL_4_-induced liver injury that receive grape seed extract nanoparticles (100 mg/kg/day) by oral gavage for four weeks. Data were expressed as mean ± S.D (*n* = 5/ subgroup). Statistical analysis was carried out by one-way ANOVA followed by post-hoc Turkeyʼs test. Values marked with the same letter are not significantly different (*p* ≥ 0.05) whereas those marked with different ones are significantly differed (*p* < 0.05)

The levels of serum ALT (74.21 ± 4.65 IU/L) (Fig. 4A), AST (62.81 ± 4.65 IU/L) (Fig. 4B) and ALP (147.41 ± 3.78 IU/L) (Fig. 4C) showed high significant elevation in the positive control rats (*p <* 0.001) after induction of CCL_4_ administration (3× higher) compared with healthy control (27.21 ± 2.17, 21.20 ± 1.92, 107.60 ± 4.28 IU/L; respectively) and vehicle control (26.41 ± 3.04, 21.41 ± 2.41, 106.2 ± 5.63 IU/L; respectively). Compared with positive control, treatment with GS extract induced significant (*p* < 0.001) amelioration of ALT, AST and ALP serum levels (47.19 ± 5.07, 40.01 ± 4.06, 126.21 ± 3.67 IU/L; respectively). While treatment based on GS extract NPs was significantly more effective than GS extract (*p* < 0.001) as it reversed CCL_4_-induced alterations in serum ALT (28.1 ± 3.40 IU/L), AST (21.4 ± 2.41 IU/L) and ALP (103.2 ± 3.34 IU/L) to levels comparable to healthy controls (no difference vs. HCs, *p* ≥ 0.05).

### Determination of in vivo effects of GS extract and GS extract NPs on oxidative stress markers of CCL_4_-induced hepatotoxicity

As shown in Fig. 4, the level of MDA (Fig. 4D) (23.8 ± 3.50 nmol/g) was significantly elevated (*p* < 0.001) in hepatic homogenate, whereas the activity of the antioxidant markers SOD (Fig. 4E) (52.36 ± 5.4 U/mg), GSH (Fig. 4F) (43.2 ± 5.2 µmol/g) and catalase (Fig. 4G) (45.6 ± 4.61 IU/g) was significantly decreased (*p* < 0.001) in CCl_4_-treated rats, as compared with the healthy control and vehicle groups. GS extract treatment promoted partial protection of the MDA (17.8 ± 1.92 nmol/g) (MDA 25% lower than CCl4-only, *p* = 0.012) with significant enhancement of SOD (72.38 ± 6.6 U/mg) and GSH (62.65 ± 6.5 nmol/g) tissue levels but the antioxidant catalase activity (48.6 ± 2.96 IU/g) was non-significantly (*p* ≥ 0.5) enhanced as compared with the positive control groups. While, considerable improvement in MDA (12.01 ± 2.34 nmol/g) (*p* < 0.001 vs. CCl_4_-treated group) (*p* = 0.999 vs. HCs), SOD (107 ± 7.38 U/mg) (*p* < 0.001 vs. CCl_4_-treated group) (*p* = 0.11 vs. HCs) and GSH (103.58 ± 4.43 µmol/g) (*p* < 0.001 vs. CCl_4_-treated group) (*p* = 0.8498 vs. HCs) with not only restored but enhanced catalase (68.6 ± 3.78 IU/g) levels was associated to GS extract NPs treatment, compared with GS extract group (*p* < 0.001).

### Determination of in vivo effects of GS extract and GS extract NPs on inflammatory markers of CCL_4_-induced hepatotoxicity

**Fig. 5:**
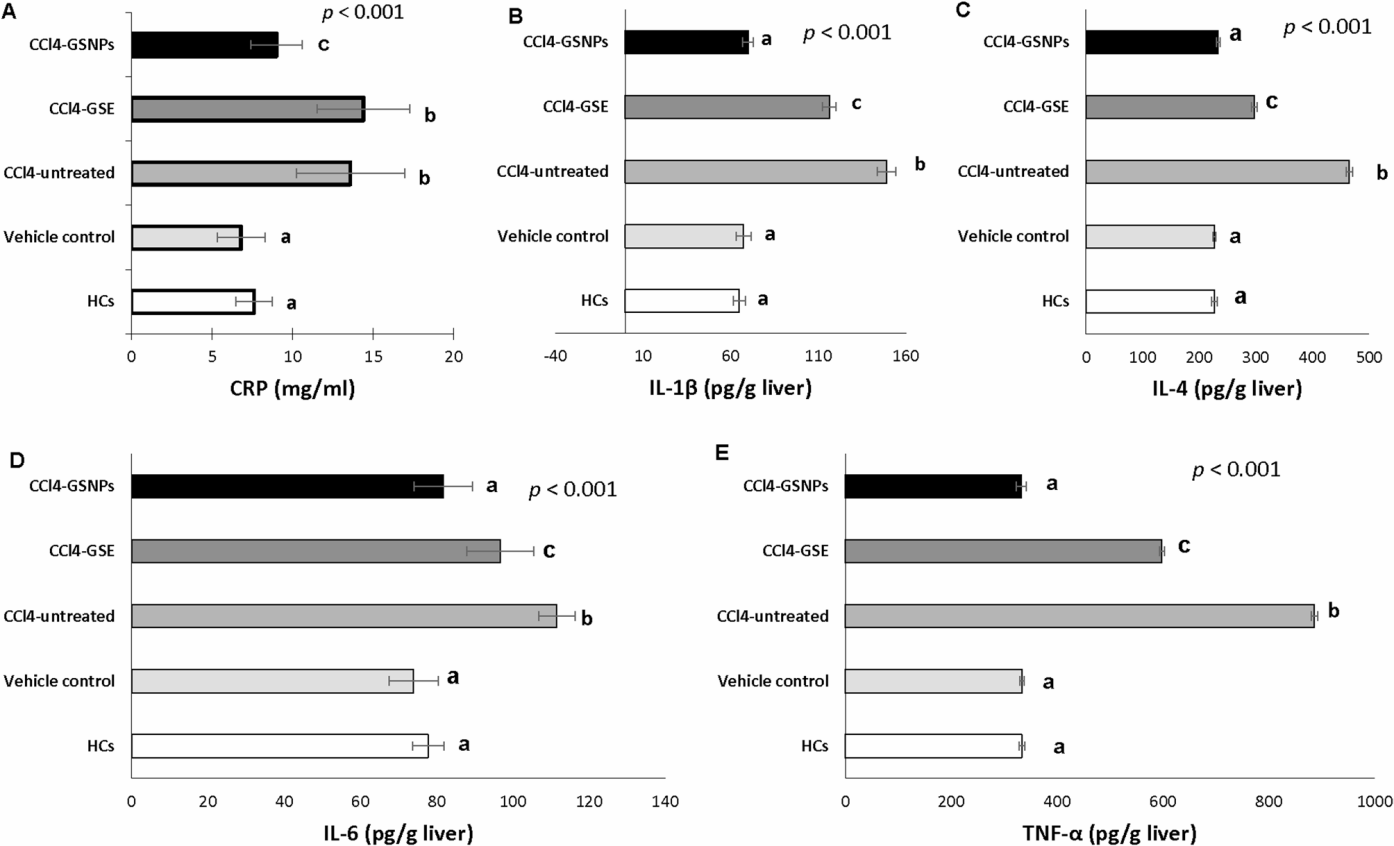
Inflammatory parameters in the different experimental groups. Levels of A] C-reactive protein (CRP) in serum, B] interleukin (IL-)1 beta (IL-1β), C] IL-4, D] IL-6 and tumer necrosis factor alpha (TNF-α) Levels in liver homogenate measured following 30 days of treatment. HCs, negative control group; vehicle control, receives olive oil only; CCl_4_-untreated, liver injury rats that receive 100 µl CCL_4_, at a dose of 0.4 ml/kg, dissolved in olive oil twice a week for three successive weeks; CCl_4_-GSE, rats with CCL_4_-induced liver injury that receive grape seed extract (200 mg/kg/day) by oral gavage for four weeks; CCl_4_-GSENPs, rats with CCL_4_-induced liver injury that receive grape seed extract nanoparticles (100 mg/kg/day) by oral gavage for four weeks. Data were expressed as mean ± S.D (*n* = 5/ subgroup). Statistical analysis was carried out by one-way ANOVA followed by post-hoc Turkeyʼs test. Values marked with the same letter are not significantly different (*p* ≥ 0.05) whereas those marked with different ones are significantly differed (*p* < 0.05)

The results in Fig. (5) showed significant increase of inflammatory cytokines in serum (CRP = 13.6 ± 3.36 mg/ml, *p* = 0.003) (Fig. 5A) and liver tissue (IL-1β = 149.1 ± 5.43, IL-4 = 465.6 ± 5.59, IL-6 = 111.6 ± 4.82 and TNF-α = 886.4 ± 5.85 pg/g) (*p* < 0.001) (Fig. 5B-E) in CCl_4_ boosted rats as compared with the healthy control.

In rats treated with GS extract, CRP non-significantly changed (14.4 ± 2.88 mg/ml) (*p* ≥ 0.05) while other inflammatory cytokines were reduced significantly (IL-1β = 116.4 ± 3.91, IL-4 = 297.8 ± 5.54, IL-6 = 96.8 ± 8.78 and TNF-α = 598.4 ± 4.82 pg/g) (*p* < 0.01) compared with the CCl_4_ positive control, but still significantly high compared with healthy rats. On the other hand, GS extract NPs-based treatment was associated with the maximal reduction of all inflammatory mediators (IL-1β = 70 ± 2.91, IL-4 = 234 ± 3.39, IL-6 = 81.8 ± 7.69 and TNF-α = 333 ± 9.66 pg/g) (*p* < 0.001) to levels analogous to healthy controls and significant from GS extract group.

## Discussion

This study aimed to compare GS extract and GS extract NPs to be used as a hepatoprotective treatment against CCL_4_-induced hepatotoxicity. Primary, GS extract was prepared vis hydroethanolic extraction method. The phytochemical composition of GS extract was elucidated using HPLC, ICP and GC-MS. HPLC analysis revealed the presence of caffeic acid, gallic acid, catechin, epicatechin and procyanidin as the major bioactive compounds. Together with our data, Sochorova et al. [64] supported the antioxidant activity of GS extract against DPPH by the richness of the total polyphenolic compounds. The presence of 14 polyphenolic antioxidant compounds in GS extract including gallic acid (the most represented), caffeic acid, coumaric acid, quercitrin, myricetin, catechin, and epicatechin were also reported in their study. In his experiment, Rockenbach et al. [65] also recorded quercetin derivatives, kaempferol derivatives, catechin (the most dominant), epicatechin and trans-resveratrol in GS extract. Oligomeric proanthocyanidins are polyphenols made up of bioflavonoid monomers, dimers and trimers are part of the basic components of GS extract. They have higher bioavailability and are potent antioxidants because of their smaller molecular size [66].

Based on the ICP analysis, calcium and potassium were detected in GS extract as the major elements in addition to magnesium, zinc and other minerals in lower concentrations. In agreement with the present results, calcium, potassium, magnesium, sodium and phosphorus were established as the mineral content of GS by ICP [67]. Al Juhaimi et al. [68] demonstrated the mineral constitution of eleven GS samples as phosphorus, potassium, calcium, magnesium, zinc and iron.

GC-MS revealed rich composition of the GS extract with aldehydes (e.g. benzoic and Cinnamon aldehyde), alcohols of terpenes (e.g. borneol, carvacrol, geraniol, linalool and α-terpineol), numerous flavonoids (e.g. myricetin, Kaempferol, catechin, epicatechin), polyphenolic acids and variety of essential (e.g. linoleic and linolenic acids) and non-essential fatty acids (hexadecenoic, Oleic and palmitic acids). The GC-MS revealed the flavonoid myricetin as the highest percentage in GS extract which have several biological properties, including anti-inflammatory, antidiabetic, anti-apoptotic and antioxidant properties [69]. Kaempferol is also identified in GS extract. Early reports supported the biological antioxidant and anti-inflammatory effects of Kaempferol through inhibition of NF-kβ activation [70], suppression of inflammatory cytokines [71] and inhibition of toll like receptor 4 (TLR 4) [72]. Elsherif et al. [73] agreed with our result and identified hexadecenoic and palmitic acids by GC-MS as a main saturated fatty acid in GS ethanolic extract. Hexadecenoic and palmitic acids can serve as efficient immunomodulators by direct action on T cells [74].

GS extract has a rich composition and multiple reported biological activities. GS extract has anti-obesity and anti-diabetic activities [75], regulation of blood lipids and improvement of microcirculation [76], anticancer and anti-inflammation role in many health problems [77] such as: inflammatory bowel disease [78], Crohn’s disease [79] and cardiovascular disease [80] but several Pharmacokinetic studies discussed their poor bioavailability if administrated orally and slow absorption through the intestine [12, 81, 82] due to their high molecular weight, low water solubility and low stability in biological fluids [83, 84]. So, it would be advantageous to develop a new method to increase GS extract-component’s oral bioavailability, and stability in the gastrointestinal system. Some previous attempts were made in this direction. Gupta and Dixit [12] enhanced GS polyphenols by their complexation with phosphatidyl choline. Castellani et al. [84] developed solid lipid nanoparticles as a delivery system in airway epithelial cells. Manca et al. [85] loaded GS extract in phospholipid vesicles for intestinal delivery. Günter et al. [86] developed Pectin-Zn-alginate hydrogel for enhanced colon-targeted GS extract delivery.

In the current study, after the phytochemical analysis of GS extract the second step was the preparation of GS extract NPs from GS powder according to a novel method early prepared in our lab. using 30% HCl solution under contentious stirring (3000 rpm) for 60 min at 30^◦^C [46]. Using the same methodology, Yehia et al. [45] prepared GS extract NPs from GS methanolic extract and suggested its enhanced antioxidant and anti-bacterial activity compared with GS extract. The formation of GS extract NPs after HCl hydrolysis of GS powder was confirmed with TEM analysis, which reveals the formation of spherical-shaped nanoparticles with good uniformity and well distributed particles without adhesion phenomenon. The particle size of the synthesized GS extract NPs is in the range of 16.5 to 22.5 nm. DLS was utilized to calculate the average particle size in solution (18.39 nm), which was smaller than earlier findings (40–70 nm) [46]. The zeta potential of GS extract NPs was highly negative (-39.42 mv), This high ZP value benefits the electrostatic repulsion between particles and increases stability [87]. By comparing the concentrations of bioactive components before and after the reaction, the synthesized GS extract NPs expressed all the bioactive components of the GS extract but in slightly lower concentrations. That could be attributed to the acidic conditions employed during synthesis. The use of 38% HCl may have contributed to partial degradation or structural transformation of sensitive polyphenolic compounds, thereby affecting their detection by HPLC. Zhang et al. [88] suggested the decreased concentrations of bioactive components as an involvement in the formation of nanoparticles as stabilizing agent, in which about 40 organic acids, phenolic compounds and alcohols of sugars were reported as examples.

The antioxidant capacity of the prepared GS extract NPs was compared with GS extract and ascorbic acid. Even with the slightly decreased concentration of the bioactive components, the antioxidant power of GS extract NPs did not modify confirming that the preparation did not modify the antioxidant power of the extract. Phenolic flavonoids, such as monomeric flavanols, dimeric, trimeric, and polymeric procyanidins, and phenolic acids, are the functional components of GS responsible for the antioxidant behavior [23, 54]. Rajakumari et al. [54] reported that high scavenging capacity via GS is via donation of hydrogen from procyanidins to the free radicals of DPPH. Procyanidins possess flavonoid moiety and functional group-OH, that have reducing action against free radicals such hydroxyl, superoxide, and DPPH alongside associated decline in the generation of hydrogen peroxide [89, 90]. Bagchi et al. [26] confirmed in vitro antioxidant capacity of GS extract that was stronger than ascorbic acid.

According to the in vitro data, GS extract NPs and GS extract modulated the coagulation process through prolongation of clotting time (PT and PTT). But the increased PT and PTT is still significantly lower than that of heparin. In consistency with our finding, Bijak et al. [91] proposed thrombin activity suppression and blood platelet response inhibition as possible mechanisms of prolonged PT and PTT due to GS extract. GS flavonoids were classified as inhibitors of platelet aggregation [92].

The analysis of normal rat hepatocytes cell viability, using MTT assay, demonstrated the absence of toxic effects of GS extract up to 783.52 µg/ml while GS extract NPs increased cell viability with higher IC50 = 1087.7 µg/m, so the conversion of the GS extract to GS extract NPs significantly enhanced their efficacy in protecting rat hepatocytes. Similarly, Aghbali et al. [93] documented the cytotoxic effect of GS extract on normal human umbilical vein endothelial cells (HUVEC) and squamous cell carcinoma (KB) cell line and reported selective cytotoxicity of GS extract to kB (IC50 245.984 µg/mL) rather than HUVEC. Montagner et al. [41] recorded no cytotoxic effect on human fibroblasts (HFF-1). Carullo et al. [94] confirmed absence of cytotoxic effect of GS extracts on RAW 264.7 cell line. The lowered cytotoxicity of GS extract NPs confirm earlier anti-cytotoxic effects of GS extract NPs reported by Farid et al. [46] and may be a result of the improved size and delivery of the antioxidant and anti-inflammatory Phytocomplexes that protect cells. Earlier studies suggested that nanoparticles-loaded GS extract confirmed enhanced cell viability by MTT assay [41, 84, 95, 96].

According to the present study, GS extract NPs have higher anti-inflammatory activity than GS extract by protecting the stability of RBCs membrane in hypotonic solution. Hemolysis is the process by which excess fluid builds up inside the cells, bursting the RBCs membrane. RBCs are more susceptible to secondary harm when their membrane is damaged [97]. Lipid peroxidation caused by free radicals ultimately causes this damage. Membrane stabilization can stop fluids and serum proteins from leaking into the tissue [98]. The highest protection capability possessed was at a concentration of 1000 µg/ml. Very limited data are available concerning the in vitro anti-inflammatory effects of GS extract and GS extract NPs and their mechanisms of action. Based on the phytochemical analysis of GS extract and GS extract NPs, the content of flavonoids may be responsible for the anti-inflammatory activity [99, 100]. The membrane of RBCs and the membrane of lysosomes are comparable. Plants that include flavonoids may be able to suppress the activity of phospholipase A2 that represents an essential component of the inflammatory process [101].

In the present study, rat model of CCl_4_-induced hepatotoxicity was designed according to Carbonari et al. [59]. The model (0.5 mL/kg, 3 weeks) (1:1 in olive oil) is a well-established subacute one for inducing measurable but non-lethal liver damage in rats Ferreira et al. [60]. Higher doses (> 1 mL/kg) risk acute liver failure, while lower doses (< 0.25 mL/kg) may not yield consistent injury [102]. Olive oil ensures slow release that prolonged hepatotoxic effects [103] and the choice of twice weekly doses for 3 weeks’ mimics chronic, progressive liver injury that allows time for Inflammation, antioxidant depletion and acute-phase response (CRP elevation) [104].

CCl_4_ is a chemical solvent that is widely utilized in industry. CCL_4_ is a well settled hepatotoxic agent that is often used to cause toxic liver damage and investigate the cellular processes underlying oxidative damages in liver due to xenobiotic induced free radical-mediated hepatotoxicity [105]. Hepatic destruction triggered by CCl_4_ is thought to develop in two stages. The first stage, which starts two hours after CCl_4_ administration, is the production of reactive radicals and oxidative stress. In tissue, CCl_4_ is converted to trichloromethyl (CCl_3_) and trichloromethyl peroxy (CCl_3_OO) free radicals which cause lipid peroxidation [106]. Increased lipid peroxidation combined with tissue antioxidant depletion results in modifications to the endoplasmic reticulum and other membrane structures, decrease in protein synthesis, loss of activation of metabolic enzymes and elevation of serum transaminase which contribute to hepatic damage [107, 108]. Furthermore, aminotransferase may be released into the bloodstream as a result of hepatocyte cellular membrane disruption that increases cell membrane permeability and facilitates cytoplasmic enzyme movement outside the cells [109]. The current data confirmed a significant increase in the levels of serum marker enzymes (e.g., AST, ALT and ALP) among the animals treated with CCl_4_. In agreement with the present findings, Teng et al. [110] suggested CCl_4_-induced hepatotoxicity was established by an increase in the levels of ALT, AST.

In the present data, Increased level of MDA in the liver tissue homogenate of rats treated with CCl_4_ indicate lipid peroxidation and damage to plasma membrane as result of oxidative stress. Raised levels of MDA following CCl_4_ administration have been well documented in various previous studies [22, 110–115]. Depletion of SOD and GSH in hepatic tissue in rats exposed to CCl_4_ indicates oxidative modifications due to increased lipid peroxidation [116, 117]. Augmented ROS level destroys membrane phospholipids, drops antioxidant levels and induce hepatic tissue damage [9]. Furthermore, catalase enzyme works to defend cells from the harmful effects of reactive oxygen species (ROS) by reducing hydrogen peroxide into water and molecular oxygen [118]. In earlier studies, hepatic cell injury is indicated by the decrease in catalase activity following CCl_4_ treatment [22, 119, 120]. The decreased CAT activity could be linked to either excessive consumption during detoxification or the oxidative inactivation or “feedback inhibition” of the enzyme’s protein due to the increased production of ROS following CCL_4_ exposure [22].

In addition to the oxidative stress, inflammation is the second critical stage influencing CCl_4_-induced liver injury as oxidant-induced activation of Kupffer cells. Macrophages represent the most prominent immune cells in the liver and are essential for preserving hepatic homeostasis as well as the basic processes involved in liver diseases [121]. Kupffer cells (KCs), resident macrophages, and monocyte-derived macrophages (MO-Mϕ_s_) make up the hepatic macrophage population [122]. In the healthy naïve liver, Resident KCs are the predominant hepatic macrophages that make up a substantial portion (80–90%) of the antigen-presenting cell (APC) population. KCs play a crucial role in preserving the liver’s immunological tolerance by stimulating regulatory T cells (Tregs) and inhibiting effector T cell activation [123]. On the other hand, MO-Mϕ_s_ detect peritoneal bacteria that invade the liver capsule and stimulate the infiltration of neutrophils to lower the levels of hepatic pathogenic load [124]. Upon the release of ROS during liver disease progression, KCs and MO-Mϕ_s_ are activated and secrete divers chemokines to recruit monocytes, other leukocytes, CD4^+^ and CD8^+^ T cells. Elevated liver pro-inflammatory cytokines (IL-1β, TNFα) with increasing levels of IL-6 promote necrosis and apoptosis of liver cells by activating caspase-3 and nuclear factor kβ (NF-κB and) COX-2 as reported in animal models of chemical-induced liver injury and this path way is called the inflammasome activation [121, 125, 126]. In addition to inflammatory cytokines, several acute phase proteins are linked to liver injury. One of these proteins is CRP, the induction and response of CRP and inflammatory cytokines intersect, since IL-6, IL-8, and TNFα can all trigger serum CRP production and release [127], but CRP can also increase the release of these cytokines at the site of local inflammation and so boost their systemic levels [128]. Our present results indicated that IL-1β, IL-4, IL-6 and TNF-α are released from macrophages in liver by CCl_4_ in association with significantly elevated serum CRP.

GS extract (200 mg/kg/day) treatment was effective to mitigate liver function enzymes MDA, SOD, GSH but has no enhancing impacts on catalase activity. Besides GS extract significantly lowered inflammatory cytokines but serum CRP was still elevated in CCl_4_-intoxicated rats. Wang et al. [18] draw attention to GS procyanidin to partially reverse the changes induced by CCl_4_ in inflammatory cytokines (TNF-α, IL-1β, IL-6, IL-17) and oxidative stress MDA markers in rat liver. Zou et al. [129] confirmed GS proanthocyanidins to protect against acute oxidative hepatotoxicity induced by CCl_4_ in mice via anti-inflammatory and antioxidant activities. GSE catechin, gallic acid and epicatechin support capability of lipid oxidation inhibition and ROS scavenging [130]. Gupta et al. [131] suggested chelating properties and effects on signal pathways and on gene expression as probable mechanisms involved in the antioxidant properties of GS extract. Ismail et al. [22] pointed to the inhibition of inducible nitric oxide synthase and xanthine oxidase gene expression as addition mechanism. Furthermore, Atasever et al. [132] suggested efficacy of GS in amelioration of CCl_4_-induced hepatocyte apoptosis and DNA fragmentation via inhibitions of caspase 3, 8 and 9 activities. The anti-inflammatory mechanism of GS extract may involve the suppression of inflammasome activation pathway by Kupffer cells in hepatocytes and parenchymal cells [133]. Subsequently, GS extract Inhibit the NLRP3, caspase-1, and IL-1β expressions due to the pre-discussed ROS elimination [134]. The inhibitory effect of GS extract on ROS/TLR4 /NF-Кβ axis is an additional probable mechanism of its anti-inflammatory potency [135]. Hamza et al. [136] proved the ability of GS extract to decrease hepatic neutrophils and macrophages proliferations in association with dramatic decrease in TNF-α and inhibited protein expressions of COX-2 and iNOS.

To the best of our knowledge, the present work is the first attempt to investigate the therapeutic abilities of GS extract NPs in case of CCl_4_-induced hepatotoxicity. The early discussed favorable properties of the GS extract are even potentiated on the nanoscale. Treatment with GS extract NPs at a dose of 100 mg/kg/day induced positive alleviating ability as presented in significant reduction of serum transaminase activity close to normality, increasing the activity of antioxidant SOD, GSH and catalase with reduction of MDA, membrane lipid peroxidation product, in CCl_4_-intoxicated rats. These results were concurrent with significant diminish of all assessed inflammatory mediators (IL-1β, IL-4, IL-6, TNF-α and CRP). GS extract NPs-based therapy was significantly more efficient compared with GS extract. Although the mechanisms for hepatoprotective activity by GSNPs is not fully assured yet. In the present findings, the nanoscale formulation of GS extract was effective in controlling the release of polyphenols, improving absorption rates and enhances the bioavailability of active components so the GS extract NPs efficiency profile was enhanced even with lower dose administration. GS extract NPs uptake may regulate the function of Kupffer cells or reduce cellular infiltration in response to reduced intrahepatic ROS and hence induce anti-inflammatory response [137], that successfully reduce the degree of liver damage in treated rats. Unfortunately, there are very limited numbers of studies on therapeutic potential of GS extract NPs on CCl_4_-induced hepatotoxicity. Abdelbaky et al. [138] indicated positive hepatoprotective ability of cellulose nanocrystals of GS than GS extract. Manca et al. [85] suggested formulations containing GS extract could improve the intestinal bioavailability of bioactive molecules ensuring a successful protection against oxidative stress and strengthen their in vivo local efficacy. Wu et al. [141] confirmed enhanced stability, improved the physicochemical and functional characteristic of nanoparticles of grape seed procyanidin.

## Conclusion

Given the present focus on natural, safe products with beneficial properties, in this work GS extract obtained from GS powder, rich in antioxidants and anti-inflammatory bioactive components, was successfully converted to GS extract NPs using 30% HCl solution. GS extract NPs seem to be an intriguing strategy for treatment of xenobiotic induced free radical-mediated hepatotoxicity and likely to attract a lot of interest in the future. GS extract NPs decreased hepatic aminotransferases, oxidative stress and proinflammatory mediators release from Kupffer cells. Further studies are essential to develop possible mechanisms and investigate GS extract NPs’ interaction with hepatocyte with special concerns to Kupffer cells.

## Data Availability

The data that support the findings of this study will be available from the corresponding author upon reasonable request.
